# Soy Isoflavones and Bone Health: Focus on the RANKL/RANK/OPG Pathway

**DOI:** 10.1155/2022/8862278

**Published:** 2022-10-25

**Authors:** Saeedeh Hosseini Hooshiar, Mohammad Tobeiha, Sadegh Jafarnejad

**Affiliations:** Research Center for Biochemistry and Nutrition in Metabolic Diseases, Kashan University of Medical Sciences, Kashan, IR, Iran

## Abstract

Bone remodels via resorption and formation, two phenomena that continuously occur in bone turnover. The RANKL/RANK/OPG pathway is one of the several mechanisms that affect bone turnover. The RANKL/OPG ratio has a substantial role in bone resorption. An imbalance between formation and resorption is related to an increased RANKL/OPG balance. OPG, a member of this system, can bind to RANKL and suppress RANK-RANKL interaction, and subsequently, inhibit further osteoclastogenesis. The serum levels of RANKL and OPG in the bone microenvironment are vital for osteoclasts formation. The RANK/RANKL/OPG system plays a role in the pathogenesis of bone disorders. This system can be considered a new treatment target for bone disorders. Soy isoflavones affect the RANK/RANKL/OPG system through numerous mechanisms. Soy isoflavones decrease RANKL levels and increase OPG levels. Therefore, isoflavones improve bone metabolism and decrease bone resorption. Soy isoflavones decrease serum markers of bone resorption and improve bone metabolism. However, while the available data are promising, the results of several studies reported no change in RANKL and OPG levels with isoflavones supplementation. In this regard, current evidence is insufficient for conclusive approval of the efficacy of isoflavones on RANKL/RANK/OPG and further research, including animal and human studies, are needed to confirm the effect of soy isoflavones on the RANKL/RANK/OPG pathway. This study was a review of available evidence to determine the role of isoflavones in bone hemostasis and the RANK/RANKL/OPG pathway. The identification of the effects of isoflavones on the RANKL/RANK/OPG pathway directs future studies and leads to the development of effective treatment strategies for bone disorders.

## 1. Introduction

Bone, as active tissue, continuously remodels throughout life [1]. Resorption and formation of bone are two phenomena that occur in the remodeling or turnover of bone and involve osteoblast and osteoclast cells. Osteoclast cells resorb the bone mineral content, whereas osteoblasts precipitate a matrix of new bone [2]. The high prevalence of osteoporosis and the increased fracture risk among the old and middle-aged can be seen due to rapid and continuous bone resorption, as opposed to growth time when bone formation surpasses bone resorption. Osteoporosis is specified by the deterioration of bone tissue microarchitecture and reduction of bone mass, which causes an enhanced risk of fracture and fragility of bone [3]. Because of the aging population of women, osteoporosis has taken the second prevalence rank among diseases. It has been considered by the World Health Organization as the main health problem in the developed world [4–6]. Approximately 30% of the cortical bone loss and around 50% of the trabecular bone loss occur during the lifetime of women. It has been reported that nearly half of bone loss occurs in the first 10 years of menopause [4]. Skeletal and extra-skeletal risk factors and age are related to osteoporotic fractures incidence [5–7]. Estrogen is involved in both the preservation of bone health and calcium homeostasis. In the postmenopausal period, the reduction of estrogen levels is associated with an increased risk of osteoporosis [8, 9]. The bone loss process is slowed using hormonal products that improve estrogen deficiency and suppress bone resorption in the first years of postmenopausal and aging [10–12]. Risks associated with hormone replacement therapy (HRT; breast cancer, venous thrombotic disease, coronary artery disease, and stroke) [13, 14] have prevented this therapy from being used as the first choice in the treatment and prevention of osteoporosis for too long. It is recommended that HRT be prescribed only for menopausal women with manifestations of modest to severe [12, 15].

Physiologically active components of soybean, such as isoflavones, seem to create specific functions of soy, including an antiobesity effect, antioxidant activity, lowering blood glucose level, and reducing osteoporosis risk. Therefore, much attention is paid to the isoflavones of soybeans that have numerous health-promoting properties [16]. The free forms of soy isoflavone, such as daidzein and genistein, with a similar structure to estradiol, can show estrogenic effects [17]. To impede the loss of bone after menopause, isoflavones have been considered an alternative to HRT to reduce the risk of osteoporosis [18–25]. The phenolic ring of soy isoflavone is the site of binding for estrogen receptors (ERs). Isoflavone affects the total body bone mineral density (BMD); however, the results are still debatable [17]. Soy isoflavones consumption increases the levels of osteocalcin, a biomarker for the formation of bone, and bone alkaline phosphatase [16], subsequently, enhances the absorption and deposition of P and Ca [26] and reduce the levels of deoxypyridinoline (DPD), pyridinoline [27], and urinary N-telopeptide [28] (Table 1). Therefore, soy isoflavone may positively modulate bone metabolism. One proposed mechanism is that soy isoflavones affect bone metabolism through the RANKL/RANK/OPG pathway. This system that has been identified over the last two decades, has increased our knowledge about bone homeostasis and metabolism [29]. The RANK\RANKL\OPG pathway controls bone metabolism by inducing osteoblast synthesis of RANKL and downregulating OPG production. Soy isoflavones affect the RANK\RANKL\OPG system through numerous mechanisms. The findings of some studies indicated that the serum RANKL/OPG ratio was decreased by isoflavones treatment [30]. But some studies did not show such an effect [17]. The current evidence cannot definitively confirm the effect of isoflavone supplementation on the RANK\RANKL\OPG system. Therefore, research into potential correlations seems relevant and significant given the significance of the RANKL/RANK/OPG pathway in bone metabolism and the potential impact of soy isoflavones on this route. We reviewed the most recent studies about this triangle among bone, soy isoflavones, and RANKL/RANK/OPG on bone health. Although current evidence is not enough for definitive approval of this efficacy, its positive responses from conducted studies are significant. Knowing more about the effects of soy isoflavones on this system directs future studies and leads to the development of effective treatment strategies for bone disorders.

## 2. Introducing the RANKL/RANK/OPG System

The RANKL/RANK/OPG pathway was first known in the mid-1990s as a substantial regulator of bone homeostasis [31]. Today we know that in addition to bone homeostasis, the RANKL/RANK/OPG system is involved in several mechanisms, such as immune regulation, the interaction between dendritic cells and T cells, lymphocyte development, mammary-gland development, certain metastatic tumors, and fever control [32]. The main components of this system include RANK (the receptor activator of nuclear factor *κ*B), RANKL (receptor activator of nuclear factor *κ*B ligand), and OPG (osteoprotegerin) [33]. RANKL, as a pivotal regulator of osteoclastogenesis, is expressed in various cells, such as osteoblasts, osteocytes, preosteoblasts, periosteal cells, dendritic cells, and vascular cells [34]. It is a ligand for RANK on the surface of osteoclasts [35]. RANKL binds to its receptor RANK, allowing the activation, survival [34], and differentiation of osteoclasts, and inhibits osteoclast apoptosis [35]. Osteoclast maturation occurs when RANK is activated by RANKL in the osteoclast [36, 37]. OPG, an atypical member of the tumor necrosis factor (TNF) superfamily, is principally expressed by the osteoblasts and bone marrow stromal cells [3, 9]. It has also been recognized on the cell membrane of lymphoid cells [33]. OPG has 7 binding domains, of which domains 1-4 mediate osteoclastogenesis inhibition and domains 5 and 6 are involved in apoptosis [34]. It is produced by different tissues, such as the intestine, lungs, kidneys, cardiovascular system (i.e., arteries, heart, and veins), and bones [34]. OPG is bound to RANKL, and subsequently, inhibits RANK activation, thus decreasing osteoclastogenesis [38].

The osteoclast differentiation, formation, and activation are suppressed by the inhibition of the RANKL/RANK pathway. Regarding this, the RANKL/OPG ratio has a substantial role in bone resorption [39]. Furthermore, an imbalance between formation and resorption is related to increased RANKL/OPG balance. In this context, the results of some studies have indicated that the ratio of RANKL and OPG levels in the bone microenvironment is vital for the regulation of osteoclasts formation. Additionally, the serum levels of RANKL and OPG are strongly involved in the pathogenesis and treatment of bone disorders [40]. Current exploration of the RANK\RANKL\OPG system has led to an increased understanding of the potential of various therapeutic modes [39].

## 3. The Role of RANKL/RANK/OPG in Bone Remodeling

Bone metabolism is a complex phenomenon that is affected not only by osteoblastic and osteoclastic activity but also by various cytokines and the balance of the RANKL/RANK/OPG pathway [17]. The signaling pathways of RANKL/RANK/OPG have been extensively investigated. Although this system is principally identified for its role in bone metabolism, it has functions in other organs and tissues. It regulates mammary-gland development, lymph-node formation, and certain metastatic tumors [29]. Based on the findings of several studies, the serum levels ratio of RANKL and OPG in the bone microenvironment is vital for osteoclasts formation and it also plays a role in the pathogenesis, prevention, and treatment of bone metabolism disorders [40].

**Figure 1:**
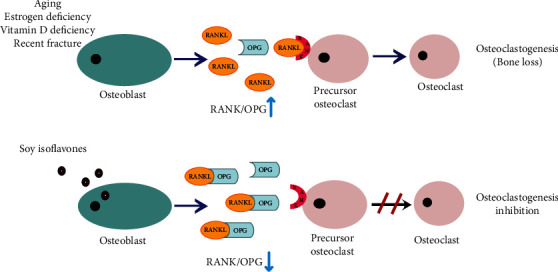
Aging, estrogen deficiency, vitamin D deficiency, and recent fracture are related to osteoporotic fractures incidence. The balance of the OPG and RANKL is vital in bone homeostasis. RANKL binds to RANK, allowing the activation osteoclasts. Mature osteoclast adheres to the bone surface and promotes bone resorption. Soy isoflavones supplementation decrease RANKL/OPG ratio by reducing RANKL and increasing OPG. OPG is bound to RANKL and subsequently inhibits RANK activation. It stops the activation of osteoclasts and decreases osteoclastogenesis. Thus the subsequent bone formation begins. (RANK: receptor activator of NF-*κ*B; RANKL: receptor activator of NF-*κ*B ligand; OPG: osteoprotegerin).

The receptor activator of nuclear factor *κ*B lacks innate protein kinase activating capacity, similar to other TNF family receptors. Therefore, it is unable to activate signaling, and adaptor molecules are needed to bind to the RANK intracytoplasmic domain [29]. These adaptors are TNF receptor-associated factors (TRAFs) that can bind to special sites in the RANK cytoplasmic domain and mediate downstream molecules. A primary step in the mediation of downstream signaling following ligation of RANKL to RANK is the TRAFs binding to RANK motifs (Motif-1, -2, or -3) on the RANK cytoplasmic domain [41]. Motif-1 activates NF-*κ*B, and subsequently, promotes the NF-*κ*B intracellular signaling cascade [41]. The NF-*κ*B translocation to the nucleus is the final step of RANK activation that regulates different genes expressions, such as the nuclear factor of activated T cells 1, c-Fos, and some bone morphogenetic proteins (BMPs) [33]. In brief, bone loss and osteoclastogenesis are promoted and increased by RANKL [40], which is a key modulator of osteoclastogenesis, and thus, mice lacking RANKL exhibited osteoporosis because of osteoclast deficiency. Maturation of osteoclasts happens when RANK is activated in the osteoclasts by RANKL that was produced by osteoblast [17]. Mature osteoclast adheres to the bone surface and promotes bone resorption by secretion of acid and lytic enzymes (e.g., cathepsin K and tartrate-resistant acid phosphatase) [29]. Osteoprotegerin is a decoy receptor from the TNF receptor family that inhibits RANK activation by RANKL, and as a result, decreases osteoclastogenesis. Several cytokines (e.g., interleukins 1 *α*, interleukins 18, and TNF-*α*), BMPs, steroid hormones (17*β* estradiol), and transforming growth factor *β* (TGF-*β*) regulate OPG expression and production [42]. Nonetheless, glucocorticoids, immunosuppressant cyclosporin A (which induces vascular disease and osteoporosis), prostaglandin E2, fibroblastic growth factor, and parathyroid hormone (PTH) reduce OPG expression. When OPG binds to RANKL, it stops the formation, survival, and activation of osteoclasts, and consequently, the subsequent bone formation begins. Since OPG tends to connect to RANKL about 500 times higher than RANK, it inhibits RANKL from binding to RANK. It prevents osteoclastogenesis and protects the bone from resorption induced by osteoclast [31]. Decreased OPG levels not only enhance osteoclastogenesis and bone resorption but also increase vascular Ca deposition. Nowadays, OPG reduction has been recognized as an independent variable for the calcification of the coronary artery [34] (Figure 1).

The balance of the OPG and RANKL expression is vital in bone metabolism and homeostasis [38]. In two disorders, namely osteoarthritis and rheumatic polymyalgia, serum OPG levels are not different from those in healthy people; however, soluble RANKL(sRANKL) levels are higher in both diseases. The results of a retrospective study on 509 patients with nonmetastatic breast cancer showed that sRANKL levels were significantly higher in patients with developed bone metastases [32]. This finding indicated that the RANK/RANKL/OPG pathway could represent a key pharmacological target in the treatment of bone metabolism disorders [34].

## 4. Function of Soy Isoflavones in Bone Homeostasis

The effect of soy isoflavones on bone metabolism indices and potential bone function of isoflavones have been investigated in previous studies [43]. Soy isoflavones can play a role in calcium metabolism and homeostasis via bone calcium mobilization to the circulation and improve serum calcium levels and physiological mechanisms related to calcium [44]. Although the mechanisms of action of isoflavones are not well understood, it seems that they not only decrease the resorption of bone but also increase the formation of bone. The increase in bone formation results from the osteoblastic activity stimulation mainly via the estrogen receptors activation. Isoflavones bind to the nuclear estrogen receptors and, since they are similar to 17*β*-estradiol, represent estrogenic activity [45]. Soy isoflavones may exhibit an osteoprotective property by a decrease of PTH levels [46], an increase of insulin-like growth factor-I secretion [47, 48], the regulation of nitric oxide production [49], inhibition of tyrosine kinase activity [50], activation of adenosine monophosphate-activated protein kinase [51, 52], upregulation of TGF-*β* [53], activation of vitamin D3 receptors [54], or antioxidant activity [55]. Soy isoflavones can also reduce bone turnover by TNF-*α* and interleukin-2 (IL-2) inhibition [56].

Interaction of soy isoflavones with serum calcium affects the total body BMD; however, not hip and spine BMD. Soy isoflavones reduce BMD and bone mineral content (BMC) of the total body at low levels of serum calcium and may increase total body BMD and BMC at high levels of serum calcium. Genistein (GEN) and daidzein, the major isoflavones in soy, have different effects on BMD. Genistein is more effective than daidzein on bone calcium mobilization, and daidzein can reduce genistein-induced BMD loss [44]. The findings of epidemiological studies showed a significant positive association between BMD and isoflavones consumption in Asian women [57]. Based on the results of a study conducted by Chen et al., although soy isoflavones could not suppress the localized loss of bone, the systemic loss of BMD was decreased [58]. Genistein can downregulate the expression of insulin-like growth factor binding protein1 messenger RNA in the eggshell gland and improve Ca homeostasis and the stability of the eggshell. It has been reported that 400 mg/kg GEN supplement increases the strength of the tibia, whereas 40 mg/kg GEN supplement ameliorates laying acting [26]. Supplemental GEN improves both the egg production and quality and the bone status of laying broiler breeder hens during the late egg-laying cycle [26]. In another study, it was found that isoflavones enhanced glycosaminoglycans content, histomorphometric indices, and mature type I collagen fibers, and subsequently improved the femur bone quality in rats. These positive effects are different in cortical and trabecular bone [57]. The results of a study indicated that the beneficial effects of isoflavones on bone are increased by folic acid supplementation, as a methyl donor [18–25].

After menopause, BMD significantly decreases and it leads to osteoporosis. This occurs due to a decrease in estrogen secretion induced by aging and a reduction in the absorbability of minerals in the intestine [27]. Soy isoflavones could imitate the influences of estrogen [59]. The bone resorption improvement can be induced by the estrogenic effects of daidzein [44]. In postmenopausal women, soy isoflavone supplements can decrease fractures risk [16]. In ovariectomized (OVX) rats, isoflavones suppress bone loss [57]. Soy isoflavones bind to estrogen receptors including ER *α* and ER *β*. Preferentially, they bind to ER *β*. It demonstrates that soy isoflavones play a role as selective modulators of estrogen receptors [26, 44, 57]. Estrogen receptor *β* has a stronger expression and wider distribution in the trabecular bone. Therefore, the spine, due to the higher trabecular bone content, is the most sensitive site for isoflavones. A meta-analysis reported that the loss of spinal bone was significantly decreased with isoflavone consumption [16]. Moreover, the results of another meta-analysis indicated that lumbar spine BMD was higher in persons that consumed isoflavones [60]. The trabecular bone volume of the femoral distal metaphysis was significantly reduced in OVX mice in 2 weeks. Nevertheless, it was improved by S-equol, a natural metabolite of daidzein, that was produced by intestinal bacteria [61]. Furthermore, the supplementation with 10 mg/day of equol significantly decreased urinary DPD, the bone resorption biomarker [62]. Bone loss induced by estrogen deficiency was enhanced by equol via the modulation of hemopoiesis and inflammatory cytokines production in cells of bone marrow [61, 63]. In OVX mice, osteoclastogenesis-mediated genes expression increased, the expression of which was suppressed by equol [61]. Perna et al. showed that no consensus was found regarding the protective effects of isoflavones (20-80 mg) and equol (10 mg) on bone resorption [64]. The results of a study by Abdi et al. indicated that isoflavones had little influence on bone health and BMD during menopause, which was inconsistent with those of some studies [65, 66]. In another study, isoflavones could decrease bone loss in women with estrogen deficiency, principally at the level of the femoral neck and lumbar spine. Likewise, the findings of a meta-analysis indicated that isoflavone had a beneficial effect on BMD of the lumbar spine, femur neck, and hip [67]. The discrepancies amongst studies can be justified by the fact that the effects of the isoflavones are higher when administered as aglycones [67]. Isoflavone-enriched whole soy milk (I-WSM) powder has approximately 8.8 times more isoflavones than usual WSM powder. Therefore, it is suggested that I-WSM is more effective than usual WSM in preventing and curing postmenopausal women with osteoporosis. Mi Kim et al. conducted a study to verify this idea [68] and reported that I-WSM seemed to be effective in the promotion of bone formation and prevention of BMD reduction [68]. The results of a study by Dasom Noh et al. showed the effective function of isoflavones plus hop prenylflavanones on the improvement of bone loss induced by estrogen deficiency [38]. The European Food Safety expressed that there “is no evidence of harm” of soy isoflavone supplement for women with post- and peri-postmenopausal status. Soy isoflavones have a safe and effective function on bone loss induced by estrogen deficiency [69]. Therefore, there is scientific evidence indicating the beneficial effects of isoflavones on bone health and the prevention and treatment of bone disorders [27, 65].

## 5. Soy Isoflavones Affect the RANKL/RANK/OPG System

**Figure 2:**
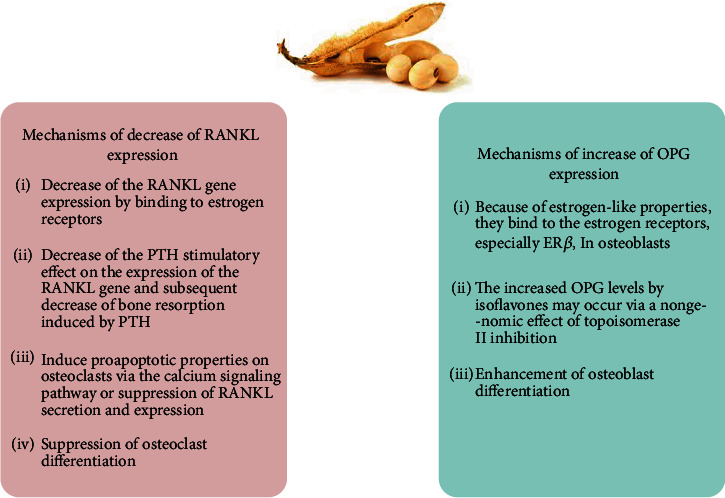
Isoflavones decrease the RANKL gene expression by binding to estrogen receptors. The PTH stimulatory effect on the expression of the RANKL gene is reduced by isoflavones and thus bone resorption induced by PTH is decreased. Decrease of RANKL levels might be induced by the proapoptotic property of the isoflavone on osteoclasts via the calcium signaling pathway or suppression of RANKL secretion and expression. The increased OPG levels by isoflavones may occur via an estrogen-mediated mechanism or a nongenomic effect of topoisomerase II inhibition. (RANK: receptor activator of NF-*κ*B; RANKL: receptor activator of NF-*κ*B ligand; OPG: osteoprotegerin).

It has been proposed that soy isoflavones affect the RANKL/RANK/OPG system through numerous mechanisms. In osteoblasts, isoflavones decrease the RANKL gene expression by binding to estrogen receptors because of their similarity to estrogen, therefore, represent estrogenic activity [28]. Additionally, the PTH stimulatory effect on the expression of the RANKL gene is reduced by isoflavones, including genistein, and consequently, bone resorption induced by PTH is decreased [35]. In genistein consumers, sRANKL serum levels decrease significantly. It might be induced by the proapoptotic property of the isoflavone on osteoclasts via the calcium signaling pathway or the suppression of RANKL secretion and expression [40]. In osteoblasts, an increase in the OPG gene expression by isoflavones has been shown in several in vitro studies [35]. The increased OPG levels by genistein may occur via an estrogen-mediated mechanism or a nongenomic effect of topoisomerase II inhibition [16]. In piglets, daidzein consumption increases the secretion of OPG and RANKL and the enhancement of osteoblast differentiation and bone mineralization are observed [70]. The increased OPG levels can decrease the RANKL interactions on the osteoclast surface, and subsequently, inhibit osteoclastic activities [71]. Soy isoflavones, especially daidzein and genistein, can activate nuclear peroxisome proliferator-activated receptors (PPAR) and mediate PPAR gene expression for the regulation of osteoclast function [72]. In addition, the reduction of bone turnover and increase of osteoblastic activity can be caused by isoflavones through IL-2 and TNF-*α* inhibition [16] (Figure 2).

The RANKL/OPG balance plays a vital role in bone resorption. The findings of a study indicated that the serum RANKL/OPG ratio was decreased by isoflavones treatment in OVX rats [30]. In another study, isoflavones plus hop prenylflavanones significantly decreased the RANKL/OPG ratio and suppressed the increase of bone turnover [38]. An in vitro study evaluated the effect of isoflavone on the RANKL/OPG pathway; accordingly, soy isoflavone tended to bond with RANK at the osteoclast, and subsequently, reduced the activation of osteoclasts in bone remodeling [73]. The results of another study indicated that isoflavone enhanced the OPG/RANKL ratio, and therefore, suppressed osteoclast differentiation indirectly [71]. Genistein aglycone is more effective compared to other current therapies in the treatment of osteoporosis, in terms of the indices of osteoblastic activity, including OPG and bone alkaline phosphatase. It also causes a better sRANKL/OPG balance. Genistein aglycone stimulates osteoblasts and suppresses osteoclasts. Furthermore, it reduces increased levels of PTH in RANKL and reverses decreased OPG expression in vitro, and consequently, antagonizes the PTH effects on bone catabolism in osteoblasts selectively [39] (Table 1). In osteopenic postmenopausal women, genistein supplementation decreased sRANKL/OPG ratio by reducing sRANKL and increasing OPG [39]. Genistein and daidzein play a role as selective modulators of estrogen receptors, especially ER*β*, and induce ER*β* binding and transcription, and subsequently, decrease osteoclastogenesis and bone resorption [74]. The results of another study showed that genistein plus vitamin D3 and calcium, along with a healthy diet, improved bone turnover in osteopenic postmenopausal women with the improvement of sRANKL/OPG balance [40]. Soy isoflavone suppresses bone loss in postmenopausal women through modulating the RANK/RANKL/OPG system [17] (Figure 1).

Some theories showed the association between the reduced estrogen levels induced by postmenopause and increased RANKL expression. In addition, the findings of another study revealed that decreased OPG level was associated with an increase in osteoclast differentiation and bone remodeling [17]. In a study, a milk product containing soy isoflavones improved bone mass and metabolism among women with postmenopausal status by increasing 25-OH-vitamin D levels and reducing bone metabolism markers [75]. The treatment with isoflavones is valuable for managing bone fragility under the conditions of reduced estrogen levels [76]. The results of another study demonstrated that the markers of bone resorption, including serum RANKL and N-telopeptide, were reduced with a daily intake of 100 mg soy isoflavone [35]. In another study, the daily consumption of 100 mg isoflavone aglycone by osteopenic postmenopausal women for 6 months had no significant effect on OPG, RANKL, and RANKL/OPG ratio. However, in comparison with the baseline, RANKL/OPG ratio and serum RANKL level were significantly reduced [17]. Treatment of osteopenic postmenopausal women with genistein significantly increases serum OPG concentrations and decreases sRANKL levels. The isoflavone genistein has a time-dependent effect and its long-term intake can lead to ongoing influences on bone homeostasis and metabolism [40]. Nonetheless, the findings of these pieces of research still cannot definitely confirm the effect of isoflavone supplementation on the RANKL/RANK/OPG system (Table 1).

## 6. Conclusions

This review summarized the role of soy isoflavones on bone hemostasis and metabolism with a focus on the RANKL/RANK/OPG pathway. The results of this study indicated that isoflavones could improve bone metabolism by decreasing RANKL and increasing OPG levels. However, while the available data are promising and conducted studies are significant in positive responses of isoflavones, several studies reported no change in RANKL and OPG levels with soy isoflavones supplementation. Therefore, current evidence is insufficient for conclusive approval of the efficacy of isoflavones on RANKL/RANK/OPG and further research, including animal and human studies, are needed to confirm the effect of isoflavones on the RANKL/RANK/OPG pathway. The identification of the effects of soy isoflavones on the RANKL/RANK/OPG pathway directs future studies and leads to the development of effective treatment strategies for bone disorders.

## Figures and Tables

**Table 1:** Characteristics of included trials.

Study name, year	Intervention form therapy and daily dosage vs. control	Sample size	Species	Follow-up duration	Significant outcome
Animal studies						
Kaludjerovic et al. [18]	2 mg daidzein × kg BW+5 mg genistein × kg BW vs. 2 mg diethylstilbestrol × kg BW (positive control) and corn oil (negative control)	81	Female and male CD-1 mice	8 months	(i) Higher femur and vertebral BMC and BMD in female mice (ii) Higher resistant to fracture in female mice (iii) Isoflavones induce protection against the bone tissue deterioration in females but not males after a decrease of sex steroid production
Ishimi et al. [19]	0.1–0.7 mg/day of genistein, 0.7–5 mg/day of genistein	10	Ovariectomized mice	4 weeks	(i) Decrease of trabecular bone loss (ii) Enhancement of BMD of the femora at a dose of 0.4 mg/day (iii) Complete restoration of bone loss at a dose of 0.7 mg/day
Picherit et al. [21]	Genistein [10 micro g/(g BW) daily], daidzein [10 micro g/(g BW) daily] vs. 17a-ethinylestradiol [30 micro g/(kg BW) daily]	65	Female Wistar rats	3 months	(i) Consumption of daidzein or 17a-ethinylestradiol was more beneficial than genistein in preventing bone loss induced by ovariectomy
Anderson et al. [22]	Genistein doses: Low (0.5 mg/d); intermediate (1.6 mg/d); and high (5.0 mg/d)		Ovariectomized, lactating rat	2 weeks	(i) Lower doses of genistein have a beneficial effect on bone (ii) High doses may have adverse effects on bone tissue potentially
Lv et al. [26]	GEN (0, 40, 400 mg/kg)	720	Laying broiler breeder	8 weeks	(i) Improvement of the eggshell strength and egg production (ii) Increase of levels of alkaline phosphatase and calcitonin in the 400 mg/kg GEN group
Noh et al. [38]	Soy-hop (0,30, 100, and 300 mg/kg)	50	Sham-operated or ovariectomized rats	8 weeks	(i) Amelioration of bone quality and metabolism
Bitto et al. [39]	Genistein aglycone (1 and 10 mg/kg s.c.); alendronate (0.003 and 0.03 mg/kg s.c.); raloxifene hydrochloride (0.05 and 0.5 mg/ kg s.c.); 17-a-ethinyl oestradiol (0.003 and 0.03 mg/ kg s.c.)	96	Ovariectomized rats	12 weeks	(i) Genistein increased both BMD and BMC, compared with the other treatments (ii) Genistein increased bone quality, breaking strength, OPG, and b-ALP (iii) Genistein reduced sRANKL and CTX
Chang et al. [54]	SIE 128.5 mg/d (genistein 8.7 mg and daidzein 3.8 m), E2 23 mg/kg, vitamin D3, soy isoflavone extract (SIE), SIE plus VitD3 and untreated group	48	Sham-operated or ovariectomized rats	14 weeks	(i) Improvement of BMD and trabecular bone volume loss (ii) SIE was more effective than 17*β*-estradiol or D3 in inhibition of increase of serum TNF-*α* levels and osteoblast osteoprotegerin expression (iii) Isoflavones prevented osteoclast proliferation (iv) Isoflavones plus D3 increased cell proliferation of cultured preosteoblasts
Santos et al. [57]	Isoflavones (80 mg/kg daily); isoflavones (200 mg/kg daily) and isoflavones (350 mg/kg daily) vs. control (treated with drug vehicle)	40	Female rats	3 months	(i) The trabecular bone volume was higher in the 350 mg isoflavones group (ii) The presence of mature type I collagen fibers and the cortical bone width were higher in the 80 mg isoflavones group (iii) Biomechanical and biophysical functions in tibias did not differ between the groups
Picherit et al. [21]	Genistein [10 mg/ (g BW daily)], daidzein [10 mg/(g BW daily)], 17a-ethinylestradiol [30 mg/kg BW daily)] vs. untreated (OVX)	65	Sham-operated or ovariectomized rats	3 months	(i) Daidzein or 17a-ethinylestradiol was more beneficial than genistein in preventing bone loss in rats
Nishide et al. [61]	0.06% (w/w) S-equol supplemented diet vs. OVX control	30	Sham-operated or ovariectomized mice	2 weeks	(i) Reduction of trabecular bone volume of the femoral distal metaphysis (ii) Equol can improve bone loss induced by estrogen deficiency
De Wilde et al. [70]	Daidzein 10−6 M, 17*β*-estradiol 10−6 M		Young female piglets	2 weeks	(i) A low dose of daidzein has anti-resorptive property by increase of the activity of porcine mature osteoblasts through ER*β*
Human studies						
Scheiber et al. [28]	3 servings daily of whole soy foods containing 60 mg of isoflavones daily	42	Normal postmenopausal women	12 weeks	(i) Decrease of risk factors of osteoporosis
Yari et al. [35]	The two tablets in the morning and evening, each 50 mg tablet contained 1.49 mg of genistein, 31.86 mg of genistin, 1.75 mg of daidzein, 13.21 mg of daidzin, 0.55 mg of glycitein and 1.14 mg of glycitin	40	Peritoneal dialysis patients	8 weeks	(i) Reduction of RANKL levels (ii) Reduction of N-telopeptide levels
Marini et al. [40]	Genistein (54 mg/d) vs. placebo	389	Postmenopausal women (age, 49–67 yr)	24 months	(i) Genistein increased OPG levels (ii) Genistein reduced sRANKL levels (iii) Genistein induced a reduction in the sRANKL/OPG ratio compared with placebo
George et al. [43]	40 g of soy or casein protein daily	90	Men and women (aged 27–87)	3 months	(i) Both soy and casein reduced bone alkaline phosphatase (ii) Both soy and casein increased serum insulin-like growth factor-I
Nayeem et al. [44]	2 pills daily. Each isoflavone pill contained 246 mg of Nova soy from [30 mg daidzein, 30 mg genistein, and 8.3 mg glycitein]. Each placebo pill contained 246 mg of a carbohydrate filler.	99	Healthy premenopausal women	2 years	(i) Genistein decreased BMD at low normal serum calcium levels (ii) Genistein increased BMD at higher serum calcium levels
Tit et al. [59]	HRT (1 mg estradiol and 0.5 mg NETA (norethisterone acetate) daily, phytoestrogens (40% standardized extract with 20 mg soy isoflavones (genistein and daidzein), twp capsules, meaning 40 mg daily vs. control group, without treatment	325	Postmenopausal women	12 months	(i) Both therapies improve bone metabolism (ii) Decrease in the evolution of bone resorption (iii) No differences between the HRT and phytoestrogens groups in BMD and bone resorption
Tousen et al. [62]	102 mg of equol daily, 6 mg of equol daily, 10 mg of equol daily vs. control	93	Postmenopausal women	12 months	(i) 10 mg/day S-equol improve bone health in postmenopausal women without adverse effects
García-Martín et al. [75]	Milk enriched with soy isoflavone (50 mg/day)	99	Postmenopausal women	12 months	(i) Increases of 25-OH-vitamin D (ii) Decrease of bone metabolism markers (iii) Soy isoflavones improve bone mass

BW: body weight; BMD: bone mineral density; BMC: bone mineral content; GEN: genistein; Soy-Hop: soy isoflavones plus hop prenylflavanones; RANK: receptor activator of NF-*κ*B; RANKL: receptor activator of NF-*κ*B ligand; OPG: osteoprotegerin; CTX: collagen type 1 cross-linked C-telopeptide; OVX: ovariectomized.
